# Allergic Rhinitis: Tailoring Immunotherapy Through Innovative Diagnostics

**DOI:** 10.7759/cureus.51370

**Published:** 2023-12-30

**Authors:** Bandar A Abushal, Abdullah Bormah, Malak Alghamdi, Yahay S Tubaigi, Amal Alomari, Safwan N Khan, Nouryah A Alhafez, Ibrahim S Aladni

**Affiliations:** 1 Department of Otolaryngology, Ministry of Health, Makkah, SAU; 2 College of Medicine, King Abdulaziz University, Jeddah, SAU; 3 Department of Family Medicine, College of Medicine, Albaha University, Al Baha, SAU; 4 Department of Medicine, Eradah Mental Health Complex, Jeddah, SAU; 5 College of Medicine, King Saud bin Abdulaziz University for Health Sciences, Jeddah, SAU; 6 Department of Medicine, Alfarabi College, Riyadh, SAU; 7 Department of Ophthalmology, King Abdulaziz University Hospital, Jeddah, SAU

**Keywords:** ar: allergic rhinitis, inflammation, molecular diagnostics, personalized medicine (pm), allergens

## Abstract

Allergic rhinitis (AR) is a chronic ailment triggered by immunoglobulin E (IgE)-mediated reactions to allergens. Generally, AR is accompanied by asthma and conjunctivitis. The risk factors of AR include both inhalant and occupational allergens and genetic factors. Although AR is not a life-threatening condition, it poses a significant risk of morbidity and hampers work-related performance. Currently, the diagnosis of AR is based on clinical history and physical examination of the patients. Furthermore, several laboratory tests such as skin pricking test (SPT), nasal allergen challenge (NAC), and computed tomography (CT) are also recommended in some cases. Nasal cytology can aid in the differentiation of rhinitis because of allergy or infection. Apart from this, molecular diagnostic modalities such as basophil activation test (BAT) and Immune Solid-Phase Allergy Chip (ISAC) can also be employed for the confirmatory diagnosis of AR. Immunotherapy has demonstrated efficacy in the management of AR, with only mild side effects. With the advancement in the diagnostic realm of AR, personalized treatment approach has also gained significant popularity. Immunotherapy is gaining evidence on becoming a personalized treatment approach for the management of AR. This article provides a comprehensive overview, aiming to bridge the gap between evolving diagnostics and personalized therapeutic strategies for allergic rhinitis.

## Introduction and background

Allergic rhinitis (AR) is one of the most prevalent conditions that afflict both children and adults. It is a major contributor to global morbidity and disability-related issues worldwide. The prevalence of AR is highly variable; however, epidemiological estimates suggest that up to 30% of adults and 40% of children are affected by AR [1]. A national cross-sectional study conducted by Alnahas et al. in Saudi Arabia reported that 5.6% of children and 14% of adults were suffering from AR. Their analysis included 3,614 children aged 6-7 years old and 4,068 adolescents aged 13-14 years old [2]. In the United States, the prevalence of AR is estimated at 7.7%. Similarly, 7.2% of children reported symptoms of AR in the past 12 months [3]. The severity of the disease is much higher during the second to fourth decade of life, after which it starts to decline. The incidence of AR is higher in high-income countries compared to middle-income and low-income countries [4]. The impact of AR is adverse on children as it hampers school performance, induces sleep problems, and limits outdoor activities [5]. It is typically referred to as an atopic disease because it is caused by reactions to inhaled allergens mediated by immunoglobulin E (IgE). In AR, the allergen-induced activation/recruitment of B cells producing IgE antibodies, mast cells, and eosinophils is triggered by type 2 helper T cells (Th2). A key feature of AR is mucosal inflammation, which is triggered by the infiltration of eosinophils and basophils [6].

Although AR is not a life-threatening ailment, complications caused by this disease can impair quality of life. The symptoms of AR begin in childhood or adolescence and continue into adulthood. Generally, there is improvement in AR symptoms slowly, and skin test reactivity tends to decline with the increasing age of the person. These symptoms are caused by the release of biochemical products in the nasal tissues after an allergic reaction. Allergic rhinitis (AR) involves two phases. The early phase begins within 20 minutes of allergen exposure. Antigen-presenting cells process allergens and present peptides on major histocompatibility complex (MHC) class II, activating naïve cluster of differentiation (CD) 4+ T cells to become allergen-specific Th2 cells. Th2 cells release cytokines (e.g., interleukin {IL} 4 and IL-13), promoting the production of allergen-specific IgE by B cells. The late phase, starting 4-6 hours post exposure, triggers nasal mucosal inflammation with the recruitment of T cells, eosinophils, basophils, neutrophils, and monocytes, driven by cytokines such as IL-4 and IL-5 [7]. When a sensitive person reencounters the allergen, it binds to the mast cell's allergen-specific IgE in the nasal mucosa, resulting in the cross-linking of high-affinity IgE receptors (FcεRI) and IgE, degranulation, and mast cell activation, and newly synthesized mediators are released. These mediators interact with nasal glands, vasculatures, and sensory nerves, resulting in acute AR symptoms [6].

## Review

Methods

A comprehensive literature review was conducted to explore the role of diagnostic methods in personalized immunotherapy among patients with allergic rhinitis, with a specific focus on its molecular diagnostics role in personalized immunotherapy. The selection of scientific articles was based on their relevance to the topic. Multiple databases, including PubMed/Medical Literature Analysis and Retrieval System Online (MEDLINE) and Web of Science, were systematically searched using keywords such as "Allergic Rhinitis," "Immunotherapy," "Personalized medicine," "Molecular diagnostics," "Diagnostic innovations," and "Allergens." While the initial search revealed more than 3,000 articles, high-quality scientific articles were considered, and each article underwent an assessment and critical evaluation prior to summarizing key findings. The review primarily focused on studies published within the last 15 years to ensure the inclusion of up-to-date research findings. The presented findings were based on studies of recent publications in the English language, specifically focusing on findings related to our topic.

Allergic rhinitis: Background about the disease

Generally, AR is also comorbid with conjunctivitis and asthma. Asthma is usually reported in 38% of patients with AR, and nasal symptoms can be observed in 6%-85% of patients with asthma [8]. The disease is characterized by nasal congestion, rhinorrhea, nasal itching, and sneezing [8]. Key risk factors identified in AR include pollens, molds, and indoor allergens such as dust mites. However, these factors are variable in different geographical regions [9]. The majority of risk factors for AR are similar to asthma and atopic dermatitis. Based on the risk factors, AR is divided into two main types: seasonal AR and perennial AR. Seasonal AR is caused by cyclical allergens including tree pollens and grass pollens. On the other hand, perennial AR occurs throughout the year and is activated by nonseasonal allergens including house dust mites and animal dander [10]. The exact diagnosis of AR is crucial for the management of this condition. Physical examination and clinical history are commonly used for diagnostic purposes. However, other diagnostic tools such as IgE testing and nasal allergen provocation can also be used for confirmatory diagnosis [6].

Currently, there are different treatment modalities available that are carried out based on the severity of AR. Failure to treat AR has serious consequences as it leads to severe complications such as asthma, respiratory tract infection, and otitis media [11]. In recent decades, there have been significant advancements in the management strategies of AR, including allergen immunotherapy (AIT) and personalized medicine. Personalized medicine is an innovative treatment approach that takes into account individual differences in patients' genes, environments, and lifestyles. However, only limited comprehensive reviews are published on AIT in AR. Therefore, the present review aims to evaluate the potential of innovative diagnostics in adjusting personalized immunotherapy for patients with AR.

Diagnostic modalities in allergic rhinitis

Due to limited public awareness, AR is often under-recognized [6]. Furthermore, AR is routinely confused with the common cold due to the limited availability of allergologists.

Standard diagnostic modalities

The diagnosis of AR depends mainly on the detailed clinical examination of the patients, regarding potential sources of exposure to allergens, seasonal symptoms, and airway hyperresponsiveness [12]. A medical history and nasal examination are the first step in diagnosing AR. Headache, facial pain, nasal itch, and prevailed sneezing are common among AR patients. Rhinoscopy assesses the absorptivity of nostrils in addition to allowing the imagining of a lower turbinate head. It also reveals the presence of nasal polyps. Furthermore, AR can also be diagnosed through skin tests such as the skin pricking test (SPT), which identifies the allergen-specific IgE bound to FcεRI molecules on the membrane of mast skin cells. Other diagnostic modalities include nasal allergen challenge (NAC) and β-transferrin evaluation from nasal fluid [13]. Generally, computed tomography (CT) scans are not needed for the diagnosis of AR, and it can be differentiated from other rhinitis by proper history, physical signs, and immunological tests such as skin prick, radioallergosorbent test (RAST), and IgE assay. The nasal allergen challenge (NAC) procedure is also a harmless and reproducible method that is well-thought-out as the gold standard for the identification of activators of AR. Different olfactory tests are performed as 20% of the AR patients suffer from smell disturbances [14]. Computed tomography (CT) can also be used to reveal chronic rhinosinusitis, tumors, or a complication when present [15]. Nasal cytology helps to differentiate between rhinitis because of allergy and infection; however, the difficulty encountered in this technique is the lack of standardization in clinical practice. When the medical treatment fails, nasal endoscopy is requested to investigate if any structural abnormalities are present. Generally, identifying the IgE sensitization to specific allergens will optimize the patient's outcome by making a personalized plan of care and implying greater diagnostic reliability for specific immunotherapy [16].

Molecular diagnostics

Molecular diagnostic modalities are also used to confirm AR. ImmunoCAP Immune Solid-Phase Allergy Chip (ISAC) is the most widely used molecular technique to quantify serum IgE (sIgE). This is a semiquantitative test that measures IgE antibodies to 176 allergen components. The Allergy Explorer (ALEX) system is also used in combination with ImmunoCAP ISAC for the molecular diagnosis of AR [17]. The sensitivity of the Allergy Explorer (ALEX) system has been at 21.05%-52.63% in allergic diseases [18]. These methods evaluate one allergen or several allergens instantaneously. These approaches have demonstrated high sensitivity and specificity and are remarkable methods for the identification of the state of the sensitization of patients, independent of their age and the drug used for their treatment [19]. The basophil activation test (BAT) is also a beneficial tool to diagnose the allergic phenotypes of rhinitis. It is known for its favorable results on clinical findings with a sensitivity of 50%-66% and specificity of >90% for different allergens. However, the sensitivity of BAT is lower than SPT, and it also triggers some false negative results [20,21]. The molecular arrangement of AR can be unraveled using BAT, and enhancing its sensitivity does not necessitate prior NAC. However, the validation of its diagnostic representation and cost-effectiveness is still being investigated [14].

New updates and innovations in allergic rhinitis diagnostics

In addition to these diagnostic tools, there are several other innovative tools for AR. Component-resolved diagnosis (CRD) is one of them. The utilization of CRD involves the characterization of molecular components associated with a specific IgE-mediated response for each allergen [22]. With traditional allergy tests, patients are tested for a mix of allergens, making it difficult to determine the exact culprit. In such cases, CRD allows for the identification of individual allergenic components, providing a clearer picture of the patient's sensitivities. This knowledge can guide personalized treatment strategies and avoid unnecessary avoidance of nonrelevant allergens. The Microtest Allergy System (Microtest Diagnostics, London, England) is also another automated microarray platform for the diagnosis of allergy [23].

In the digital age, mobile applications and wearable devices are being developed to help individuals track and manage their AR-related symptoms. This information can be shared with healthcare professionals, facilitating more targeted treatment plans. These tools allow users to monitor allergen exposure, track medication usage, and receive personalized allergy alerts. There are mobile applications such as Allergy Monitor, MASK-air, and Pollen that are useful for AR patients [24]. Telemedicine is also an innovative tool for AR diagnosis and self-management. Telemedicine is transforming healthcare delivery, including AR diagnostics. Video appointments enable healthcare providers to make a preliminary diagnosis and discuss treatment options, all within the convenience of the patient's home. Through remote consultations, patients can discuss their symptoms and medical history and undergo virtual examinations [25].

Immunotherapy in allergic rhinitis

Allergen-specific immunotherapy (ASIT) is a promising and the only therapeutic approach for dealing with seasonal AR. This therapy affects all the pathological mechanisms of sensitivity, offers protective effects, and also provides a complete resolution after the end of AR treatment. Immunotherapy modifies the immune response to allergens, leading to reduced symptom severity and long-term benefits. The medical effects of the AIT comprise the fall of symptoms, reduction in the demand for antiallergenic agents, avoidance of allergen-spectrum extension, and respiratory asthma in AR patients. It also improves the quality of life of AR patients [26].

Available options

Allergen immunotherapy (AIT) can be administered in either sublingual (sublingual immunotherapy {SLIT}) or subcutaneous (subcutaneous immunotherapy {SCIT}) ways. The inoculation of SCIT in patients is done in increasing quantities every week for many weeks. This therapy is followed by once-a-month maintenance doses for almost three years. The completion of SCIT is done at a specialized allergy hospital that has all the equipment to handle severe anaphylaxis. Furthermore, SLIT has appeared to be a substitute route of desensitization in AR, and it comprises daily tablets or drops made of allergen extracts that are sited under the tongue of the patients. This therapy can be completed by the patients themselves at their home and hence offers an added favor and safe profile over the SCIT [10]. Test administration routes are effective, safe, and tolerative even after treatment cessation. Adaptive and innate immune reactions that are contributors to sensitized inflammation are repressed by AIT [27].

The mechanism of action of immunotherapy in AR involves several steps. These include the identification of allergens, allergen desensitization, immune system modulation, and anti-inflammatory effects. The eventual objective of immunotherapy in AR is to achieve long-term tolerance to allergens [27]. The success of immunotherapy involves various alterations in the immune system. This is mainly linked with elevated levels of regulatory T cells (Tregs), which consequently leads to an increase in IL-10 and transforming growth factor-beta (TGF-β), the shifting of Th2 to Th1 immune responses, and a reduction in Th2 cells. Apart from this, there is also an increased level of serum immunoglobulin G4 (sIgG4)-blocking antibodies that can inhibit mast cells. In SLIT for AR, an increase in serum immunoglobulin A (sIgA) levels is also observed [26]. This whole cascade of reaction starts when an allergen is inhaled. In the first step, epithelial cells recruit dendritic cells, polarizing them to pro-allergic dendritic cells type 2 (DC2) phenotypes. Then, these cells present them to naïve T cells, which help the development of Th2. Ultimately, these Th2 cells help the maturation of B cells, leading to allergen-specific IgE production [10].

In most cases, AIT is well-tolerated and safe in both children and adults. However, in some cases, it can produce side effects that are mostly mild or local in nature [28]. Several reported side effects of immunotherapy include asthma, urticaria, angioedema, anaphylaxis, and other local and systemic side effects. Some of the local effects of SLIT include oropharyngeal and gastrointestinal symptoms, which are observed in almost 50%-85% [29]. Other SCIT-related adverse reactions include local pain, needle phobia, and issues with lymphatic drainage in postmastectomy patients [28].

Personalized medicine of immunotherapy

Personalized medicine is an approach to tailor an individual's specific characteristics, including their genetic makeup, immune response, and environmental factors. This approach has enhanced the precision of allergy diagnosis, high performance with personalized treatments, and the efficiency of allergen immunotherapy. According to European Forum for Research and Education in Allergy and Airway Diseases (EUFOREA)-Allergic Rhinitis and its Impact on Asthma (ARIA)-European Position Paper on Rhinosinusitis and Nasal Polyps (EPOS)-Integrated Care Pathways for Airway Diseases (AIRWAYS ICP) statement, endotype-driven treatment should be incorporated as personalized treatment in the management of AR [30]. Personalized medicine in the immunotherapy of allergic rhinitis is still an area of active research and development, and there is ongoing exploration of new techniques and technologies to further enhance the effectiveness and precision of treatment. However, personalized medicines for allergies are probable to face trials, for instance, growing heterogeneity and complexity, therapeutic wondering, and effective management of patients with uncontrolled symptoms [31]. Allergic diseases are well-suited for personalized medicine. There are different tools such as proteomics, transcriptomics, epigenomics, and breathomics. Such tools can enhance the efficacy of allergen immunotherapy. A major challenge to the implementation of precision medicine is high costs associated with molecular analyses and biological treatments in personalized medicine. However, many fundamental principles of personalized medicine can already be integrated into first- and secondary-level management [31].

Regarding precision medicine, AIT is one of the best examples of personalized therapy for the cure of AR. It brings sensitized immune forbearance by improving several regulatory cells to regulate type 2 inflammation. The effectiveness of AIT/ASIT is reported for improving allergic symptoms, dropping allergen reactivity, decreasing medicine requirements, avoiding asthma development, and refining the quality of life of AR patients. Conventional SCIT has some drawbacks that include necessitating some doses frequently, clinic visits, systemic allergic reactions, and high costs. The multiple administration routes of AIT provide an alternative and aid in improving the compliance and safety of the patients. Novel biological and innovative tools are being developed for added improvement in the efficacy of AIT [32].

## Conclusions

In conclusion, the landscape of diagnosing and treating allergic rhinitis has evolved significantly, with a particular emphasis on immunotherapy. The journey through the intricacies of allergic rhinitis, from standard diagnostic modalities to cutting-edge molecular diagnostics, showcases the expanding arsenal available to healthcare professionals. The emergence of personalized medicine in allergic rhinitis marks a transformative shift toward more targeted and effective interventions. The clinical implications of these advancements are profound. However, the research on this topic is still limited. Therefore, future studies should focus on immunotherapy as a treatment modality for allergic rhinitis.
